# Malignant catarrhal fever induced by *Alcelaphine herpesvirus 1 *is characterized by an expansion of activated CD3^+^CD8^+^CD4^- ^T cells expressing a cytotoxic phenotype in both lymphoid and non-lymphoid tissues

**DOI:** 10.1186/1297-9716-42-95

**Published:** 2011-08-22

**Authors:** Benjamin G Dewals, Alain Vanderplasschen

**Affiliations:** 1 Department of Infectious and Parasitic Diseases, Immunology-Vaccinology (B43b), Faculty of Veterinary Medicine, University of Liège, B-4000 Liège, Belgium

## Abstract

*Alcelaphine herpesvirus 1 *(AlHV-1) is carried by wildebeest asymptomatically. It causes a fatal lymphoproliferative disease named wildebeest-derived malignant catarrhal fever (WD-MCF) when cross-species transmitted to a variety of susceptible species of the *Artiodactyla *order. WD-MCF can be reproduced experimentally in rabbits. In a previous report, we demonstrated that WD-MCF induced by AlHV-1 is associated with a severe proliferation of CD8^+ ^T cells in the lymphoid tissues. Here, we further studied the mononuclear leukocytic populations in both the lymphoid (throughout the infection and at time of euthanasia) and non-lymphoid (at time of euthanasia) organs during WD-MCF induced experimentally in rabbits. To reach that goal, we performed multi-colour flow cytometry stainings. The results obtained demonstrate that the development of WD-MCF correlates in peripheral blood with a severe increase of CD8^+ ^cell percentages; and that CD3^+^CD8^+^CD4^- ^T cells were the predominant cell type in both lymphoid and non-lymphoid organs at time of euthanasia. Further characterization of the mononuclear leukocytes isolated from both lymphoid and non-lymphoid tissues revealed that the CD8^+ ^T cells express high levels of the activation markers CD25 and CD44, produce high amount of gamma-interferon (IFN-γ) and perforin, and showed a reduction of interleukin-2 (IL-2) gene expression. These data demonstrate that the development of WD-MCF is associated with the expansion and infiltration of activated and cytotoxic CD3^+^CD8^+^CD4^- ^T cells secreting high amount of IFN-γ but low IL-2.

## Introduction

Malignant catarrhal fever (MCF) has been described as a fatal lymphoproliferative disease of a variety of species of the *Artiodactyla *order that includes cattle. The main causative agents of MCF are two closely-related gammaherpesviruses included in the newly defined *Macavirus *genus, *Ovine herpesvirus 2 *(OvHV-2) and *Alcelaphine herpesvirus 1 *(AlHV-1) [1]. These viruses cause no apparent disease in their natural host species. Sheep are naturally infected by OvHV-2 which is responsible for the sheep-associated form of MCF (SA-MCF) when cross-species transmitted to susceptible hosts such as cattle. Wildebeest (*Connochaetes taurinus*) carry AlHV-1 responsible for the wildebeest-derived form of MCF (WD-MCF) [2,3]. In sub-Saharan Africa, cross-species transmission of AlHV-1 to susceptible host species occurs throughout wildebeest grazing areas and largely affects cattle. WD-MCF has an important impact on the herds grazing in these regions [4,5]. In addition, WD-MCF has also been reported throughout the world in zoological collections where mixed artiodactyls including wildebeest are kept [6]. Experimentally, WD-MCF can be induced in rabbits. Based on clinical signs, macroscopical and histopathological observations, the disease induced by AlHV-1 in rabbits is undistinguishable to the disease observed in the naturally susceptible species, which make rabbits reliable as an experimental model [3,7-10].

WD-MCF is characterized by a combination of lymphoproliferation and degenerative lesions caused by unknown mechanisms [3,11,12]. The infiltration of lymphoid cells surrounding small arteries and veins in virtually all organs is a typical lesion observed in WD-MCF. Though the disease and the lesions observed in SA-MCF and WD-MCF are similar, it is unknown whether the pathogenesis of both forms is similar. Single-colour flow cytometry stainings on cells from cattle and rabbits developing WD-MCF revealed a severe increase of CD8^+ ^and T cells in the lymphoid organs [13,14], while immunohistochemistry analyses showed that the infiltrating cells in non-lymphoid organs were mainly composed of T cells, CD4^+ ^and CD8^+ ^cells [10,14,15]. Similar findings were also observed in animals developing SA-MCF [10,16]. Though these results suggested that WD-MCF is associated with an accumulation of T cells including CD8^+ ^T cells, single-colour analyses are not sufficient to precisely define the cellular population present in lymphoid and non-lymphoid tissues and to determine whether they display a similar phenotype in both type of tissues.

Cell-mediated cytotoxicity via natural-killer cells or activated CD8^+ ^T cells has been proposed to explain the degenerative lesions associated with MCF [3,11,17]. In a recent study, we demonstrated that WD-MCF is associated with a severe proliferation of CD8^+ ^T cells in lymphoid tissues such as peripheral blood mononuclear cells (PBMC), lymph nodes and spleen and that at least 10% of these cells carry a latent infection [13]. It is unknown whether these CD8^+ ^T cells display an activated phenotype compatible with the cytotoxic role that they could play in WD-MCF pathogenesis. Supporting this hypothesis, T cell activation and cytotoxicity markers were upregulated in a microarray study on lymph node cells isolated from SA-MCF clinical cases [18]. However, the activated cell population(s) has(have) not been clearly identified nor the proportion of activated cells amongst the defined phenotype(s).

In the present study, we pursued our investigation on the pathogenesis of WD-MCF induced by AlHV-1 in rabbits. We aimed to determine the main population changes in mononuclear leukocytes and their activation status in both lymphoid and non-lymphoid tissues after AlHV-1 infection. We demonstrated that the prominent mononuclear leukocytic population present in both lymphoid and non-lymphoid tissues during WD-MCF are CD3^+^CD8^+^CD4^- ^T cells displaying an activated and cytotoxic phenotype. In the light of these data, we discussed the mechanisms by which activated CD8^+ ^T cells could play a role in the pathogenesis of WD-MCF.

## Materials and methods

### Cell line and virus strain

Bovine turbinate fibroblasts (BT, ATCC CRL-1390) were cultured in Dulbecco's modified Essential Medium (D-MEM, Invitrogen, Paisley, United Kingdom) containing 10% foetal calf serum (FCS) (Lonza, Basel, Switzerland). The pathogenic AlHV-1 C500 strain isolated from an ox with MCF [19] was used throughout this study and maintained by limited passage (< 5) in BT cells.

### Induction of MCF in rabbits

Specific pathogen-free New-Zealand white rabbits were purchased from Harlan Nederland and housed individually throughout this study. Two groups, each comprising three rabbits were used. Animals of each group were inoculated intravenously with 4 × 10^6 ^mock-infected BT cells, or with 4 × 10^6 ^BT cells infected with the AlHV-1 C500 WT strain, respectively. Infected cells for inoculation were harvested from cultures in which the cytopathic effect reached 90% or more. Rabbits were examined daily for clinical signs and body temperature. According to bioethical rules, rabbits were euthanized when rectal temperature remained higher than 40°C for two consecutive days.

### Ethics statement

The animal study performed has been accredited and approved by the local Ethics Committee of the University of Liège (Belgium) and received the reference number 1127.

### Ex vivo cell suspension preparation

PBMC were isolated from 10 mL of blood collected from the ear central artery just before infection and at different time-points post-infection. Immediately after euthanasia, single-cell suspensions were prepared from popliteal lymph node (pLN), spleen, liver, lung and kidney as follows. For each organ, a fragment of 500 mg was delicately chopped in sterile RPMI-1640 (Invitrogen) containing 5% FCS and passed through a 70 μm cell-strainer. Mononuclear leukocyte suspensions were prepared from peripheral blood and single-cell suspension derived from organs with Ficoll-Paque Premium density gradient media (GE Healthcare, Uppsala, Sweden). Briefly, 10 mL single-cell suspension was diluted 1:1 in sterile PBS, then loaded on 8 mL Ficoll-Paque density cushion and centrifuged (1825 × *g*) for 20 min at room-temperature. Mononuclear leukocytes at the interface were collected and washed twice in ice-cold PBS before further analysis.

### Immunofluorescent surface staining of rabbit cells for flow cytometry analysis

Washes and incubation steps were performed in FACS buffer (PBS pH7.4, 0.1% BSA, 0.05% NaN_3_). (*i) Detection of CD4, CD8, IgM, CD14 and pan T marker expression*. Cells were incubated with mAb anti-rabbit CD4 (KEN-4, IgG2a, AbD Serotec, Oxford, United Kingdom), CD8 (12.C7, IgG1, AbD Serotec) and IgM (NRBM, IgG1, AbD Serotec) for 10 min on ice. After washing, cells were incubated for 10 min on ice with isotype-specific PE-conjugated rat anti-mouse IgG1 (A85-1, BD Biosciences, Erembodegem, Belgium) and biotinylated rat anti-mouse IgG2a (R19-15, BD Biosciences) antibodies. After an additional wash, cells were incubated with FITC-conjugated anti-rabbit T cells (KEN-5, IgG1, AbD Serotec), Pacific Blue-conjugated anti-human CD14 (TÜK-4, IgG2a, AbD Serotec) and APC-conjugated streptavidin (BD Biosciences). (*ii) Detection of CD25 and CD44 expression in CD4^+ ^cells*. Cells were incubated with mAb anti-rabbit CD25 (KEI-ALPHA1, IgG2b, AbD Serotec), mAb anti-rabbit CD44 (W4/86, IgG1, AbD Serotec) and mAb anti-rabbit CD4 (KEN-4, IgG2a, AbD Serotec) for 10 min on ice. After washing, cells were incubated for 10 min on ice with isotype-specific PE-conjugated rat anti-mouse IgG1 (A85-1, BD Biosciences), FITC-conjugated rat anti-mouse IgG2b (R12-3, BD Biosciences) and biotinylated rat anti-mouse IgG2a (R19-15, BD Biosciences) antibodies. After an additional wash, cells were incubated with APC-conjugated streptavidin (BD Biosciences). *(iii) Detection of CD25 and CD44 expression in CD8^+ ^cells*. Cells were incubated with mAb anti-rabbit CD25 (KEI-ALPHA1, IgG2b, AbD Serotec) and mAb anti-rabbit CD44 (W4/86, IgG1, AbD Serotec) for 10 min on ice. After washing, cells were incubated for 10 min on ice with isotype-specific PE-conjugated rat anti-mouse IgG1 (A85-1, BD Biosciences) and biotinylated rat anti-mouse IgG2b antibodies (R12-13, BD Biosciences). After an additional wash, cells were incubated with FITC-conjugated anti-rabbit CD8 (12.C7, IgG1, AbD Serotec) and APC-conjugated streptavidin (BD Biosciences). Staining specificity was controlled with the appropriate isotype-matched antibody controls (IgG1, MOPC-31C; IgG2a, G155-178; IgG2b, MPC-11; BD Biosciences). Compensation was performed on single-stained samples before acquiring the multi-colour stainings. Before flow cytometry analysis, cells were incubated with 0.5 μg/mL 7-aminoactinomycin D (7-AAD, Sigma, St Louis, MO, USA) to exclude dead cells.

### Intracellular staining

*(i) Detection of CD3 and perforin*. Mononuclear leukocytes isolated from PBMC or organs were incubated in FACS buffer with FITC-conjugated anti-rabbit CD8 (12.C7, IgG1, AbD Serotec) and washed. Cells were then fixed for 20 min on ice in PBS 2% paraformaldehyde and then permeabilized for 30 min in 0.1% saponin permeabilization buffer. Finally, rabbit cells were stained with cross-reacting PE-conjugated anti-human CD3ε chain (CD3-12, rat IgG1, AbD serotec) or PE-conjugated anti-human perforin (δG9, mouse IgG2b, BD Biosciences). Anti-CD3ε antibody binds to a conserved cytoplasmic epitope (ERPPPVPNPDYEPC, Accession number: AB035151) present on the ε-chain of the CD3 co-receptor in several species, including human, bovine and rabbit [20]. *(ii) Detection of gamma interferon (IFN-γ)*. Mononuclear leukocytes were cultured at a concentration of 10^6 ^cells/mL in RPMI media containing 10% FCS, 2 mM L-glutamine, 25 mM Hepes, 0.1 mM non essential amino acids, and 1 mM Na Pyruvate (Invitrogen). Cells were incubated 4 h in presence or absence of 20 ng/mL phorbol 12-myristate 13-acetate (PMA), 1 μg/mL ionomycin and 10 μg/mL brefeldin A (Sigma). At the end of the incubation period, cells were harvested and surface staining performed. Cells were incubated in FACS buffer with anti-rabbit CD8 (12.C7, IgG1, AbD Serotec), then washed and incubated with biotinylated rat anti-mouse IgG1 antibody (A85-1, BD Biosciences). After a final wash cells were incubated with FITC-conjugated anti-rabbit T cells (KEN-5, IgG1, AbD Serotec) and APC-conjugated streptavidin (BD Biosciences). Following surface staining, cells were fixed and permeabilized as described above. Finally, rabbit cells were stained with cross-reacting PE-conjugated anti-bovine IFN-γ (CC302, AbD serotec).

### CD8^+ ^cell purification

Single**-**cell suspension of mononuclear leukocytes isolated from PBMC, peripheral lymph nodes and spleen were stained with FITC-conjugated anti-rabbit CD8 (12.C7, IgG1, AbD Serotec) before enrichment using anti-FITC MACS beads and LD columns according to the manufacturer's instructions (Miltenyi Biotec, Bergisch Gladbach, Germany). CD8^+ ^cells were further purified using a high-speed cell sorted (FACSAria, BD Biosciences) before RNA isolation. The obtained purity was always higher than 96%.

### RNA isolation and real-time PCR

Total RNA was purified using RNeasy Miniprep kit (Qiagen, Hilden, Germany) with on-column DNAse-I treatment. RNA concentration was measured on a Nanodrop-1000 (Thermo Scientific, Wilmington, DE, USA). cDNA was reverse-transcribed from 100 ng RNA with oligo-dT and Improm-II™ Reverse Transcription System (Promega, Madison, WI, USA). Real-time PCR was performed using iQ SYBR Green reaction mix (Biorad, Hercules, CA, USA) and the reactions run on an iCycler system (Biorad). Primers used are listed in Table 1. Standard curves were generated using specific PCR amplicons or rabbit multi-gene plasmid (kindly provided by Dr S. Lukehart).

**Table 1:** Oligonucleotides used for PCR reactions

Sequence	Primer	Primer sequence	Amplicon (bp)	Reference
*Cd8b*	RbCD8-Fwd	5'-TTGGCATGGGGGTGGAAAAG-3'	231 bp	[48]
	RbCD8-Rev	5'-GGAACCGGCACACTCTCTT-3'		
*Prf1*	RbPRF1-Fwd	5'-CAGTACAGCTTCAACACGGAC-3'	176 bp	[49]
	RbPRF1-Rev	5'-ATGAAGTGGGTGCCATAGTTG-3'		
*Faslg*	RbFASLG-Fwd	5'-ACACCTATGGAATTGCCCTGG-3'	312 bp	[50]
	RbFASLG-Rev	5'-TGACCAGTGATATCTCAGAGAC-3'		
*Ifng*	RbIFNg-Fwd	5'-TTCTTCAGCCTCACTCTCTCTG-3'	224 bp	[51]
	RbIFNg-Rev	5'-TGTTGTCACTCTCCTCTTTCC-3'		
*Tnf*	RbTNFa-Fwd	5'-GTCTTCCTCTCTCACGCACC-3'	335 bp	[51]
	RbTNFa-Rev	3'-TGGGCTAGAGGCTTGTCACT-3'		
*Il2*	RbIL2-Fwd	5'-TGAAACATCTTCAGTGTCTAGAAG-3'	203 bp	[51]
	RbIL2-Rev	5'-CCATTTGTTCAGAAATTCTACAATG-3'		
*Il10*	RbIL10-Fwd	5'-GAGAACCACAGTCCAGCCAT-3'	179 bp	[51]
	RbIL10-rev	5'-CATGGCTTTGTAGACGCCTT-3'		

### Flow cytometry

Flow cytometry acquisitions and cell sorting were performed using a three-laser Becton Dickinson fluorescence-activated cell sorter (FACSAria). Data were analyzed with FlowJo software v7.5.5 (Treestar, Ashland, USA).

### Statistical analysis

Statistical analyses were conducted using GraphPad Prism 5 software (La Jolla, USA). One-way ANOVA test was used to determine significant differences, with Bonferroni's multiple comparison post-test applied to calculate significance values between samples.

## Results

### Phenotypic analysis of rabbit peripheral blood mononuclear cells by multi-colour flow cytometry

**Figure 1:**
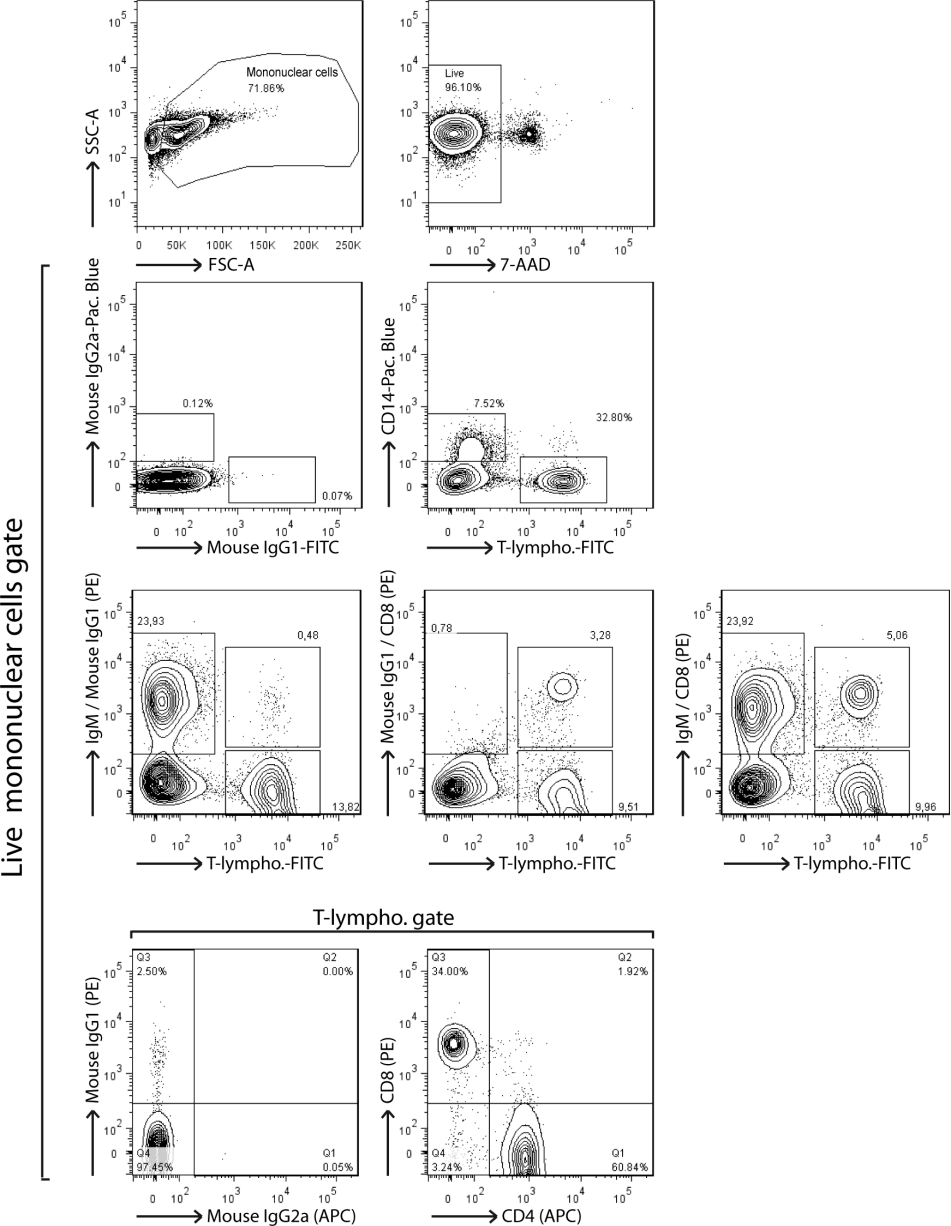
**Analysis of rabbit peripheral blood mononuclear cells by multi-colour flow cytometry**. PBMC were isolated from K_3_EDTA-sampled blood of naive rabbits before five-colour flow cytometry analysis. Specific detection of monocytes, B cells and T cell subsets consisted in a 3-step staining procedure. PBMC were first stained with anti-rabbit IgM (or mouse IgG1 isotype control), anti-rabbit CD8 (or mouse IgG1 isotype control) and anti-rabbit CD4 (or mouse IgG2a isotype control). Stainings were revealed with PE-conjugated rat anti-IgG1 or biotinylated rat anti-IgG2a, as secondary staining. Final staining was performed with streptavidin-APC, FITC-conjugated anti-rabbit T lymphocytes (or FITC-conjugated mouse IgG1 isotype control) and anti-human CD14 (or Pacific Blue-conjugated mouse IgG2a isotype control). Live lymphocytes were gated on 7-AAD^- ^cells.

As opposed to the extensively studied mouse model, in-depth studies of rabbit cellular responses are rendered complex due to the lack of available specific antibodies. Here, we devised an original strategy using multi-colour flow cytometry to further investigate the cellular responses in rabbit PBMC (Figure 1). The described five-colour staining strategy resulted in the detection of the main cellular subsets of PBMC such as B cells, monocytes, and CD4^+/- ^or CD8^+/- ^T cells in a single sample.

### Induction of WD-MCF in rabbits

**Figure 2:**
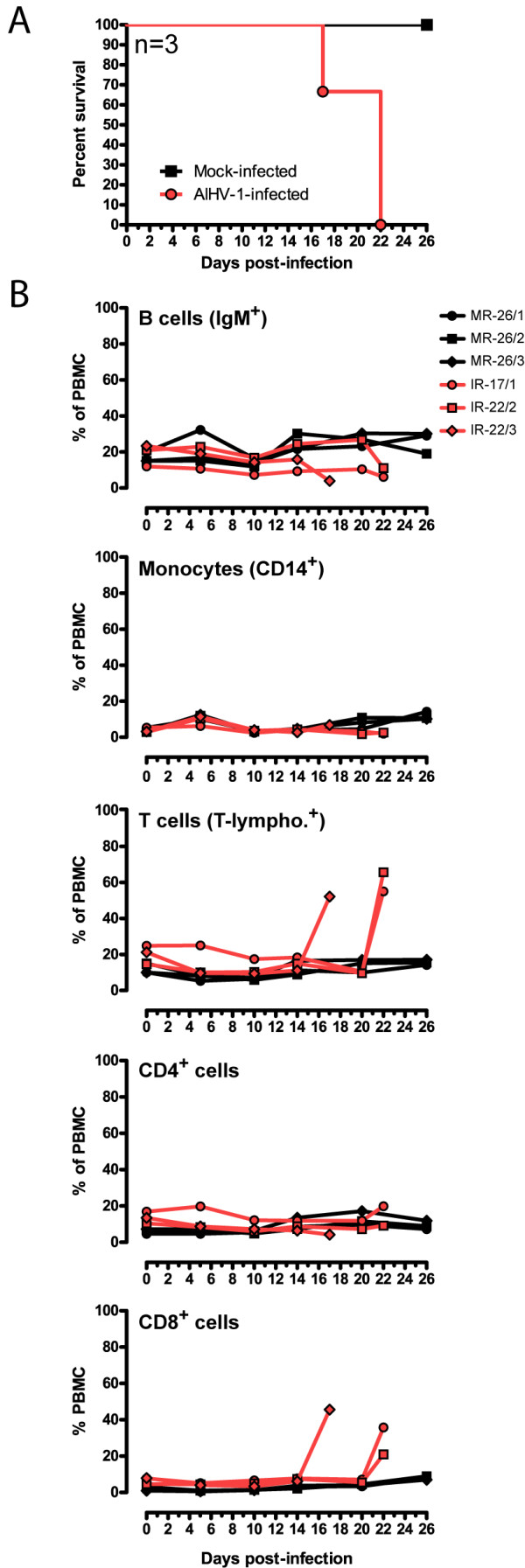
**Induction of WD-MCF in rabbits**. On day 0, two groups, each consisting of three rabbits, were inoculated intravenously with 3 × 10^6 ^mock-infected or AlHV-1-infected BT cells. Rabbits developing WD-MCF were euthanized when rectal temperature remained higher than 40°C for two consecutive days. Rabbits were identified according to their infected (IR) or mock-infected (MR) status and according to the time of euthanasia (see numbers). (A) Survival curve of AlHV-1-infected rabbits. (B) Relative percentages of B cells (IgM^+^), monocytes (CD14^+^), T cells (T-lymphocytes^+^), CD4^+ ^and CD8^+ ^cells in PBMC isolated from both groups every 4-5 days after infection, stained and analyzed by flow cytometry as described in Figure 1. Data are representative of two independent experiments (*n *= 3).

In order to reproducibly induce WD-MCF and investigate the cellular responses following AlHV-1 infection in the light of our previous findings [13], two groups of three rabbits were mock-infected or infected intravenously with the pathogenic AlHV-1 C500 strain [19]. Rabbits infected with AlHV-1 developed persistent hyperthermia associated with palpable enlargement of pLN at 21.33 ± 6.02 days post-infection (pi) (Figure 2a). Rabbits infected with AlHV-1 developed the expected clinical signs such as prostration, anorexia, adypsia and hyperthermia. Necropsy revealed the characteristic gross lesions and cellular infiltrates in liver, lung and kidney (data not shown) [21,22]. Mock-infected rabbits remained healthy throughout the experiment course and were euthanized at day 26 pi.

### Phenotypic characterization of mononuclear leukocytic populations in lymphoid and non-lymphoid organs during WD-MCF in rabbits

**Figure 3:**
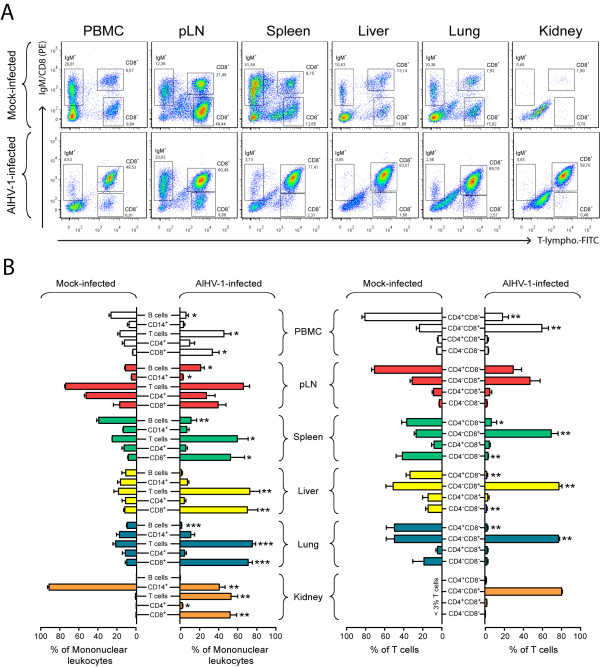
**CD8^+^CD4^- ^T cells are the prominent cellular phenotype in lymphoid and non-lymphoid organs during WD-MCF in rabbits**. At time of euthanasia, analysis by multi-colour flow cytometry was conducted on PBMC and mononuclear leukocytes isolated from pLN, spleen, liver, lung and kidney of mock-infected or AlHV-1-infected rabbits developing WD-MCF. (A) Flow cytometry dot plots obtained by multi-colour staining of PBMC and mononuclear leukocytes isolated from pLN, spleen, liver, lung and kidney of one representative rabbit of each group at time of euthanasia. (B) Relative percentages of B cells (IgM^+^), monocytes (CD14^+^), T cells (T-lymphocytes^+^), CD4^+ ^T cells and CD8^+ ^T cells in the lymphocyte gate are shown on the left panel. Relative percentages of CD4^+^CD8^-^, CD4^-^CD8^+^, CD4^+^CD8^+^, CD4^-^CD8^- ^T cells are shown on the right panel. The percentages of T-lymphocytes-positive cells in the kidney of mock-infected animals were below 3% and were therefore not plotted. Data are representative of two independent experiments (*n *= 3). Bars show means ± SEM. *p < 0.05, **p < 0.01, ***p < 0.001.

**Figure 4:**
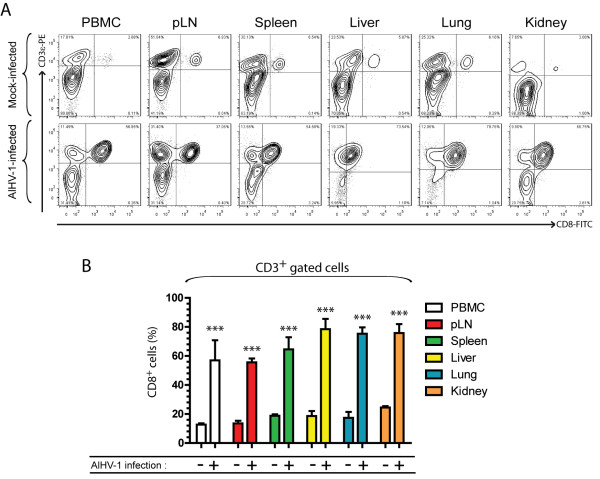
**Co-expression of CD3 by CD8^+ ^cells in lymphoid and non-lymphoid organs during WD-MCF in rabbits**. At time of euthanasia, analysis by multi-colour flow cytometry was conducted on PBMC and mononuclear leukocytes isolated from pLN, spleen, liver, lung and kidney of mock-infected or AlHV-1-infected rabbits developing WD-MCF. (A) Flow cytometry contour plots obtained by multi-colour staining of PBMC and mononuclear leukocytes isolated from pLN, spleen, liver, lung and kidney of one representative rabbit of each group at time of euthanasia. (B) Relative percentages of CD8^+ ^cells in gated CD3^+ ^cells are shown. Data are representative of two independent experiments (*n *= 3). Bars show means ± SEM. ***p < 0.001.

AlHV-1 infection did not cause significant change in B cells (IgM^+^), monocytes (CD14^+^) or CD4^+ ^cell frequencies in PBMC over time (Figure 2b). We however observed a severe increase of CD8^+ ^cells in the PBMC of all AlHV-1-infected rabbits after appearance of clinical signs, consistent with our previous observations [13]. To further investigate the cellular responses in lymphoid and non-lymphoid tissues of rabbits developing WD-MCF clinical signs, mononuclear leukocytes were isolated from peripheral blood, pLN, spleen, liver, lung and kidney of mock- or AlHV-1-infected rabbits. We then analyzed the cellular composition using the multi-colour staining as described above. A severe disruption of the cellular composition was observed in mononuclear leukocytes isolated from all tissues of AlHV-1-infected rabbits (Figure 3). In lymphoid tissues, the cell composition in the spleen was the most affected, with significant reduced IgM^+ ^and CD4^+ ^cell population frequencies and severe increased percentages of CD8^+ ^T cells, compared to mock-infected rabbits. The proportions of CD8^+ ^T cells in liver, lung and kidney were also strongly increased, with CD8^+^CD4^- ^T cells representing nearly the totality of infiltrating mononuclear leukocytes (Figure 3b). Although the KEN-5 clone was used based on its description as a pan-T cell marker of rabbit, the specificity of this antibody has not been fully identified [23]. To determine whether the CD8^+ ^cells expanding during WD-MCF are really T cells, we used an anti-human CD3 cross-reacting antibody raised against an intra-cytoplasmic peptide of the CD3ε chain. Co-staining of mononuclear leukocytes from lymphoid and non-lymphoid tissues with anti-CD8 and CD3ε demonstrated that all CD8^+ ^cells also co-expressed the CD3 co-receptor (Figure 4). Though previous studies reported the presence of T cells and CD8^+ ^cells in WD-MCF lesions [10,14,15], our results demonstrate that the systemic subacute periarteriolitis observed in WD-MCF is mainly composed of CD3^+^CD8^+^CD4^- ^T cells.

### Activation status of CD8^+ ^T cells in lymphoid and non-lymphoid organs during WD-MCF in rabbits

**Figure 5:**
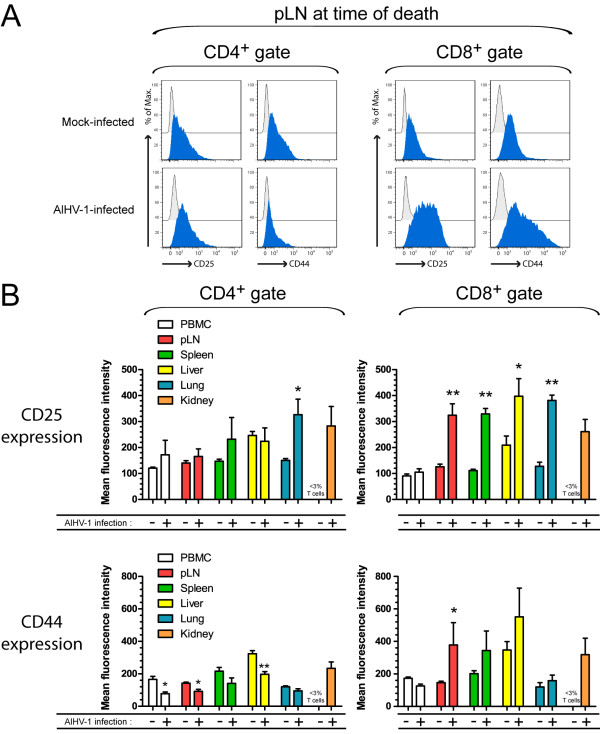
**CD25 and CD44 expression in CD8^+ ^T cells isolated from lymphoid and non-lymphoid organs during WD-MCF in rabbits**. At time of euthanasia, analysis by multi-colour flow cytometry was conducted on PBMC and mononuclear leukocytes isolated from pLN, spleen, liver, lung and kidney of mock-infected or AlHV-1-infected rabbits developing WD-MCF. (A) Half-offset histograms of CD25 or CD44 expression by CD4^+ ^(left panel) or CD8^+ ^cells (right panel) in mononuclear leukocytes isolated from pLN of one representative rabbit of each group. Data were obtained by triple-staining of CD4 or CD8 with isotype control (grey histograms) or CD25 and CD44 (blue histograms) as described in Methods. (B) Mean fluorescence intensities of CD25 and CD44 in CD4^+ ^(left panel) or CD8^+ ^cells (right panel) obtained by multi-colour flow cytometry analysis. The percentages of CD4^+ ^or CD8^+ ^cells in the kidney of mock-infected animals were below 3% and were therefore not plotted. Data are representative of two independent experiments (*n *= 3). Bars show means ± SEM. *p < 0.05, **p < 0.01.

T cell activation results in the over-expression of the interleukin 2 receptor (CD25) in the majority of species, including rabbits [24]. Upregulation of H-CAM (CD44) in association with the downregulation of the L-selectin (CD62L) has also been described as a signature of T cell activation in different species [25]. Though CD25- and CD44-specific monoclonal antibodies have been developed for rabbits, no reagent for specific detection of rabbit CD62L is currently available. We used a multi-colour flow cytometry strategy to determine whether the expression of the two cell surface markers CD25 and CD44 was altered during WD-MCF. Mononuclear leukocytes were isolated from different tissues as described above to analyze the expression of CD25 and CD44 in both CD4^+ ^and CD8^+ ^T cells (Figure 5). We observed that CD25 expression was not significantly altered in CD4^+ ^T cells, except in the lung (Figure 5b). CD44 expression in these cells was however reduced in most of analyzed tissues; including PBMC, pLN and liver. In contrast, we observed a strongly increased expression of CD25 in CD8^+ ^T cells isolated from pLN, spleen and liver. CD44 expression was significantly increased in CD8^+ ^T cells isolated from the pLN, but the expression of CD44 was not significantly modified in the other analyzed tissues of rabbits developing WD-MCF clinical signs. Together, these results demonstrate that CD8^+ ^infiltrating the lesions during WD-MCF display an activated phenotype during WD-MCF with strong upregulation of CD25.

### Production of gamma interferon by CD8^+ ^T cells in lymphoid and non-lymphoid organs during WD-MCF in rabbits

**Figure 6:**
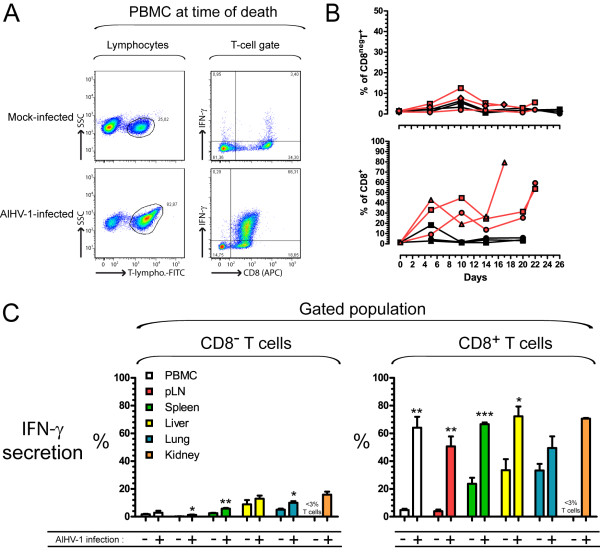
**IFN-γ production by CD8^+ ^T cells isolated from lymphoid and non-lymphoid organs during WD-MCF in rabbits**. At time of euthanasia, analysis by multi-colour flow cytometry was conducted on PBMC and mononuclear leukocytes isolated from pLN, spleen, liver, lung and kidney of mock-infected or AlHV-1-infected rabbits developing WD-MCF. Cells were cultured with PMA/ionomycin and brefeldin A for 4 h and analyzed for ex vivo IFN-γ production capability by triple-colour staining. (A) Flow cytometry dot plots obtained by multi-colour staining of pLN cells of one representative rabbit of each group. A first gate was placed on T-lymphocytes (left column) to measure IFN-γ production in CD8^- ^or CD8^+ ^T cells (right column). (B) IFN-γ production in CD8^- ^or CD8^+ ^T cells in PBMC over time after infection. Data represent individual measurements. (C) IFN-γ production in CD8^- ^or CD8^+ ^T cells at time of euthanasia in PBMC and mononuclear leukocytes isolated in pLN, spleen, liver, lung and kidney. The percentages of T-lymphocytes-positive cells in the kidney of mock-infected animals were below 3% and were therefore not plotted. Data are representative of two independent experiments (*n *= 3). Bars show means ± SEM. *p < 0.05, **p < 0.01, ***p < 0.001.

Upon activation, CD8^+ ^or CD4^+ ^T cells can secrete IFN-γ, an effector cytokine having major functions in the clearance of intracellular pathogens. To further characterize the T cell activation after AlHV-1 infection, the production of IFN-γ by CD8^+ ^or CD8^- ^(mainly CD4^+^) T cells was determined (Figure 6). Single-cell suspensions were cultured in presence of phorbol 12-myristate 13-acetate (PMA), ionomycin and brefeldin A before being stained for cytokine secretion. Following gating on T cells (Figure 6a), the percentages of IFN-γ-producing CD8^+ ^or CD8^- ^T cells were analyzed at regular intervals in PBMC after AlHV-1 infection (Figure 6b). The results showed that CD8^- ^T cells did not exhibit significant increased secretion of IFN-γ (Figure 6b, upper panel), whereas CD8^+ ^T cells produced large amount of the cytokine as early as 5 days pi with up to 80% of CD8^+ ^T cells secreting IFN-γ at time of euthanasia (Figure 6b, lower panel). After development of WD-MCF clinical signs, mononuclear leukocytes were isolated from lymphoid and non-lymphoid organs as described above, and restimulated with PMA/ionomycin in presence of brefeldin A for intracellular IFN-γ detection. Again CD8^- ^T cells did not show substantial increased cytokine secretion whereas more than 50% of the CD8^+ ^T cells isolated from PBMC, pLN, spleen, liver and kidney of infected rabbits secreted IFN-γ (Figure 6c). Together, these results demonstrate a strong activation of CD8^+ ^T cells during WD-MCF.

### Perforin expression by CD8^+ ^T cells in lymphoid and non-lymphoid organs during WD-MCF in rabbits

**Figure 7:**
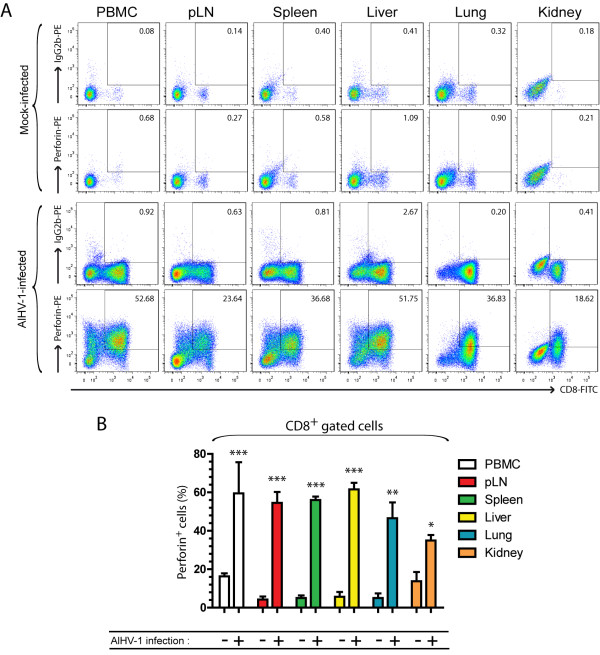
**Perforin production by CD8^+ ^T cells isolated from lymphoid and non-lymphoid organs during WD-MCF in rabbits**. At time of euthanasia, analysis by multi-colour flow cytometry was conducted on PBMC and mononuclear leukocytes isolated from pLN, spleen, liver, lung and kidney of mock-infected or AlHV-1-infected rabbits developing WD-MCF. (A) Flow cytometry dot plots obtained by multi-colour staining of PBMC and mononuclear leukocytes isolated from pLN, spleen, liver, lung and kidney of one representative rabbit of each group at time of euthanasia. (B) Perforin production in CD8^+ ^cells at time of euthanasia in each lymphoid and non-lymphoid selected tissue. Data are representative of two independent experiments (*n *= 3). Bars show means ± SEM. *p < 0.05, **p < 0.01, ***p < 0.001.

To further investigate the potential of CD8^+ ^T cells expanding during WD-MCF to be cytotoxic in vivo, we determined the production of perforin in these cells by intracellular staining (Figure 7). The results showed that perforin was mainly produced in CD8^+ ^T cells in all lymphoid and non-lymphoid tissues and that these cells produced large amount of the cytotoxic protein with up to 82% of CD8^+ ^T cells of PBMC being positive at time of euthanasia (Figure 7b). Together, these results suggest that CD8^+ ^T cells during WD-MCF have an important cytotoxic potential in vivo.

### Gene expression profile of CD8^+ ^T cells during WD-MCF in rabbits

**Figure 8:**
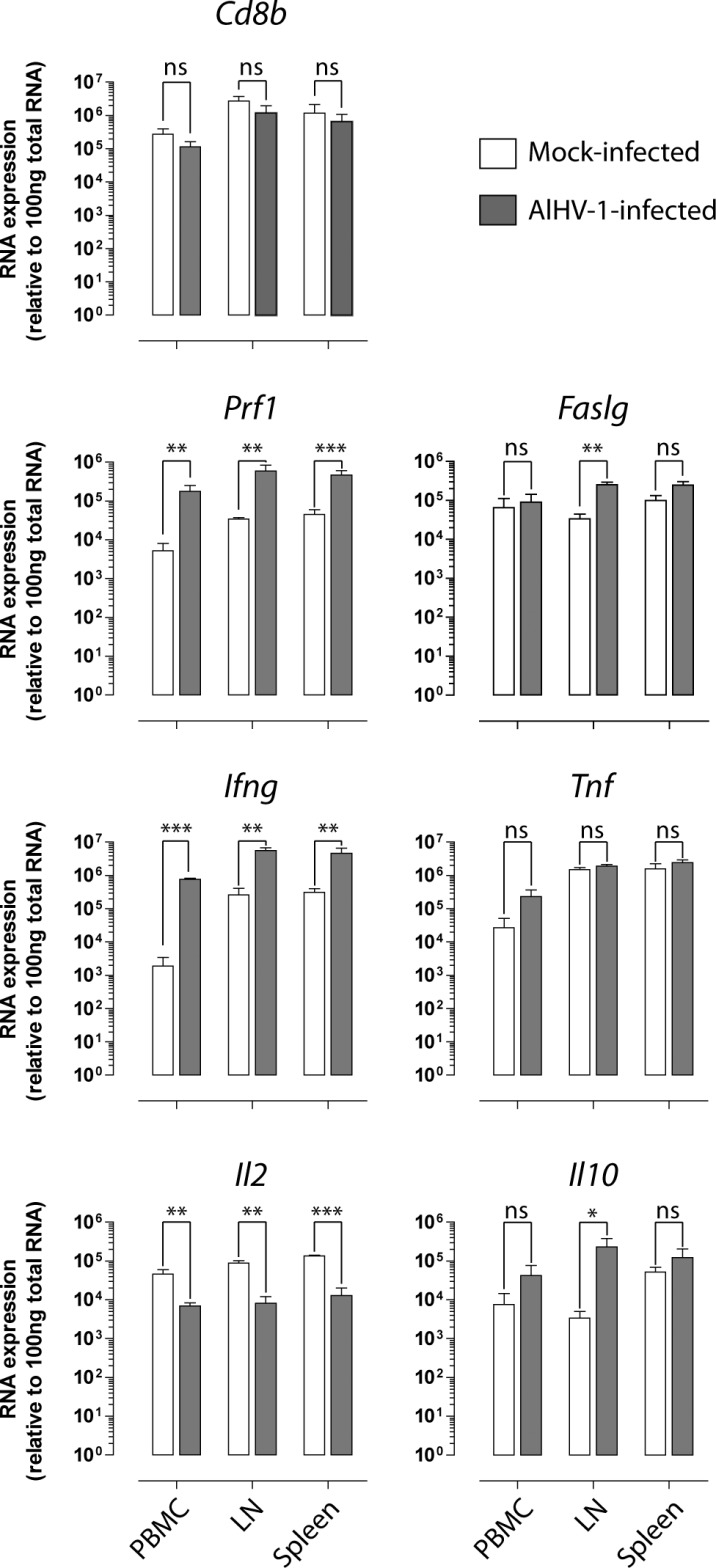
**Gene expression profile of CD8^+ ^cells during WD-MCF in rabbits**. (A) CD8^+ ^cells were sorted to high purity (≥ 96%) and RNA extracted before analysis of expression levels of genes associated with T cells and cytotoxicity: CD8-beta chain (*Cd8b*), perforin (*Prf1*), FAS-ligand (*Faslg*), IFN-γ (*Ifng*), tumor necrosis factor-alpha (*Tnf*), interleukin 2 (*Il2*) and interleukin 10 (*Il10*). Relative expression levels normalized to 100 ng total RNA are shown. Data are representative of two independent experiments (*n *= 3). Bars show means ± SEM. *p < 0.05, **p < 0.01, ***p < 0.001, ns: not significant.

To better characterize the expanding CD8^+ ^T cells during WD-MCF, these cells were purified (≥ 96%) from PBMC, LN and spleen and analyzed by real-time PCR for relative expression levels of selected genes related to cytotoxicity and cytokines (Figure 8). CD8^+ ^cells from non-lymphoid organs were not analyzed due to the very low number of mononuclear leukocytes that could be recovered from these tissues in mock-infected animals. Gene expression of the CD8-beta chain was similar in CD8^+ ^cells purified from mock- and from AlHV-1-infected animals, indicating that *Cd8b *gene expression is not affected by AlHV-1 infection and further confirming the equivalent purity of CD8^+ ^cells isolated from both groups. We have shown in Figure 7 that perforin is highly expressed in CD8^+ ^cells during WD-MCF. These results were confirmed as we observed significantly increased perforin mRNA expression levels in CD8^+ ^cells during WD-MCF. However, FAS-ligand expression levels were not significantly increased, except in LN, suggesting that cell death induction through this pathway is not upregulated in CD8^+ ^cells during WD-MCF. Cytokine gene expression level analyses in CD8^+ ^cells showed that TNF-α and IL-10 mRNA expression levels were not affected by AlHV-1 infection, except a significant upregulation of IL-10 in LN. Importantly, AlHV-1 infection resulted in a strong upregulation of IFN-γ gene expression in CD8^+ ^cells, as observed at the protein level in Figure 6. Finally, IL-2 mRNA expression levels were 3.5 to 7 times lower in CD8^+ ^cells from AlHV-1-infected compared to mock-infected rabbits.

## Discussion

In the present study, we used multi-colour flow cytometry stainings to phenotype mononuclear leukocytes in both lymphoid and non-lymphoid tissues after AlHV-1 infection of rabbits. Using these stainings, we demonstrated that the prominent cell population present in lymphoid and non-lymphoid tissues during WD-MCF consists of CD3^+^CD8^+^CD4^- ^T cells that express an activated and cytotoxic phenotype revealed by high expression of CD25, as well as increased IFN-γ and perforin production.

How AlHV-1 infection induces the development of WD-MCF is still a matter of a considerable debate, mainly due to the lack of readily available tools in cattle or in the rabbit experimental model. Upon infection, rabbits develop a disease (clinical signs, gross lesions and histopathological lesions) that is undistinguishable to the disease developed by susceptible species such as cattle [3,7-10,13]. Based on these data, this small animal has been extensively used as a model of choice to better understand the pathogenesis of WD-MCF. AlHV-1 transmission to susceptible species mainly occurs through the nasal route [26-28]. Though nasal inoculation of rabbits with low-passage cell-free viral particles leads to the development of WD-MCF, AlHV-1 is mainly cell-associated in cell culture and rapidly loses its pathogenicity after several passages which render more difficult to obtain pathogenic cell-free virus [29,30]. Intravenous administration of cell-associated AlHV-1 is however more reproducible with much lower variability in the incubation period before WD-MCF induction in rabbits (authors' unpublished data). In term of clinical signs and lesions, both inoculation routes lead to a similar disease.

We used multi-parameter flow cytometry stainings in rabbits in order to conjointly detect CD14-, IgM-, T-lymphocyte marker-, CD4- and CD8-expressing mononuclear leukocytes. The specificity of the rabbit T-lymphocyte marker was also further confirmed by cross-reacting anti-human CD3 intracellular staining. Such staining procedure allows for repeated analyses of the variations within the main cell populations over time in PBMC as well as in lymphoid or non-lymphoid tissues at time of euthanasia after AlHV-1 infection. Our results demonstrated that WD-MCF in rabbits is associated with the expansion of CD3^+^CD8^+^CD4^- ^T cells, representing the prominent cell population in both lymphoid and non-lymphoid tissues, such as liver, kidney and lung. Most of CD8^+ ^T cells from lymphoid (except PBMC) and non-lymphoid organs expressed high levels of CD25 in animals developing WD-MCF (Figure 4). Importantly, these observations were however not observed in CD4^+ ^T cells. In addition, most of CD8^+ ^T cells secreted IFN-γ demonstrating that this cell population display a strong activation status. The percentages of IFN-γ-positive cells amongst CD8^- ^T cells (mainly CD4^+ ^T cells) remained low throughout the course of the experiment confirming that the activation was restricted to CD8^+ ^T cells. The severe increased of IFN-γ gene expression was also confirmed by real-time RT-PCR on sorted CD8^+ ^cells from rabbits developing WD-MCF. Increased IFN-γ production by expanding CD8^+ ^T cells was accompanied by their high expression of perforin, demonstrated both by intracellular staining and real-time RT-PCR. Upregulation of granzymes, perforin and IFN-γ has been recently reported in lymph nodes of cattle developing SA-MCF [18] and CD8^+ ^perforin^+ ^γδ-T cells were identified in the perivascular spaces during experimental SA-MCF in bison [31]. Together with the results of these two recent studies in SA-MCF, the present study suggests that the pathogenesis of WD-MCF and SA-MCF is similar and relies on the expansion of activated cytotoxic CD8^+ ^T cells.

CD8^+^CD4^- ^T cells infiltrating the perivascular spaces in most organs during WD-MCF are cytotoxic, activated and secrete IFN-γ in vivo. Though it remains unknown whether these cells are responsible for the degenerative lesions and the death of infected animals, their observed phenotype strongly suggests that these cells could play a major role in the pathogenesis of WD-MCF. Large-granular lymphocyte (LGL) expressing CD8 and/or CD4 were produced by extensive ex vivo cultures of recombinant IL-2-stimulated lymphoid cells isolated from animals developing WD- or SA-MCF [12,32]. These established LGL cell lines were able to mediate killing of cells irrespective of their infection status or the homologous MHC of the target cell [32]. However, the phenotype of these LGL cell lines as well as their activation status might not reflect what occurs in vivo but could result from their selective expansion in culture after activation by IL-2. Moreover, we observed severely reduced IL-2 gene expression in sorted CD8^+ ^cells from animals developing WD-MCF (Figure 8) and in the all pLN tissue (data not shown). Reduced IL-2 transcripts in LN was also recently reported during SA-MCF by microarray analyses [18], suggesting that the use of this cytokine as growth factor is probably not the best choice to mimic ex vivo the expansion of CD8^+ ^cells that occurs during WD-MCF. Further, our results demonstrate that the low abundance of IL-2 transcripts in all LN during SA- or WD-MCF is not related to the loss of IL-2 producing cells but rather to the downregulation of the cytokine transcription in CD8^+ ^cells. The observation that genetically IL-2-deficient mice develop with age fatal and uncontrolled lymphocyte proliferation [33,34] has led to the hypothesis that IL-2 impairment could play a central role in the pathogenesis of MCF [18]. In this context, the proliferation of activated cytotoxic CD8^+ ^T cells could be due to the inhibition of IL-2 expression by AlHV-1 latent infection of CD8^+ ^T cells leading either to the loss of autocrine "self-control" of T cell proliferation or to an indirect inhibition of regulatory T cells (Treg) homeostasis [35,36]. Future studies on the role of IL-2 and Tregs during AlHV-1 infection are required to address these questions.

Cyclosporin-A treatment of rabbits infected with OvHV-2 did not impair SA-MCF development though infiltration of the perivascular spaces was no longer observed [37], this observation suggests that intense cell proliferation is not essential for a fatal outcome in MCF. In addition, hyper acute forms of MCF result in sudden death without the development of detectable degenerative lesions [3]. These findings support the need of further experiments to unravel the putative role of activated CD8^+ ^T cells and cell-mediated cytotoxicity in the pathogenesis of WD-MCF. We observed that most of CD8^+ ^T cells at time of euthanasia produced IFN-γ, a pro-inflammatory cytokine. SA-MCF-derived LGLs were also shown to produce IFN-γ, tumor necrosis factor alpha, interleukin-4 and 10 [32]. The cause of sudden death in MCF could therefore be a hypercytokinaemia syndrome. Though future experiments should test this hypothesis, no significant increase of TNF-α or IL-10 gene expression could be observed in sorted CD8^+ ^cells (Figure 8).

We showed that IFN-γ-producing CD8^+ ^T cells in PBMC increased progressively and peaked just before the death of infected animals. At that particular time point, viral infection is predominantly latent and at least 10% of CD8^+ ^T cells carry the viral genome [13]. Latent AlHV-1 infection of CD8^+ ^T cells could therefore be responsible for their deregulation resulting in uncontrolled activation and proliferation. As suggested before [13], proliferation of CD8^+ ^T cells due to their latent infection would result in clonal expansion of primarily infected cells, in which case the expanding infected cell population should display a homogenous phenotype in all tissues. This hypothesis is further supported by our observation that the cells present in lymphoid and non-lymphoid tissues are composed of a homogenous CD8^+^CD4^- ^T cell population producing IFN-γ and perforin. Moreover, bioluminescent imaging of organs explanted from rabbits infected with a recombinant AlHV-1 strain expressing a luciferase reporter protein demonstrated that viral infection was widely distributed in lymphoid and non-lymphoid organs such as liver, kidney and lung [38]. Human and animal gammaherpesviruses have acquired various mechanisms to evade major histocompatibility complex (MHC) class I-mediated killing of infected cells by CTLs [39-43]. Immune evasion of CTL-mediated killing by AlHV-1-infected CD8^+ ^T cells might therefore contribute to viral persistence. In gammaherpesvirus infection, some viral evasion of CTL confers a prominent role in host defence on CD4^+ ^T cells. During infection with murid herpesvirus 4 (MuHV-4), CD4^+ ^T cells antiviral effector function has been demonstrated to be CD8-independent but dependent on IFN-γ secretion [44]. CD4^+ ^T cells during AlHV-1 infection might consequently represent a key effector cell population to mediate viral clearance and protection. Though Sparks-Thissen and colleagues have shown that IFN-γ was necessary to mediate CD4^+ ^T cell dependent killing [44], it is unknown whether this effector cytokine have anti-viral function during WD-MCF. IFN-γ has been shown to be essential to inhibit reactivation from latency [45], and infection of IFN-γ-receptor deficient mice led to B-cell lymphoproliferative disease in MuHV-4 infection [46,47]. We showed in the present study that most of the expanding CD8^+ ^T cells produce IFN-γ. In the light of the results obtained from MuHV-4 infection studies, it is possible that though not being able to mediate cellular-mediated killing of the infected cells, IFN-γ produced by infected CD8^+ ^T cells could inhibit reactivation of AlHV-1 from latency. Virus infection of CD8^+ ^would lead to their uncontrolled activation and indirectly evade recognition through a putative IFN-γ-mediated inhibition of reactivation. Future *in vivo *neutralization experiments to investigate whether IFN-γ plays a role in the inhibition of viral lytic gene expression, allowing AlHV-1 genome to be latently maintained in infected cells are under progress.

In this study, we characterized the phenotype of mononuclear leukocytes in lymphoid and non-lymphoid tissues of rabbits developing WD-MCF. We demonstrated that the majority of mononuclear leukocytes present in lymphoid and non-lymphoid tissues, such as liver, lung and kidney, are CD3^+^CD8^+^CD4^- ^T cells over-expressing activation surface markers and that most of these cells produced IFN-γ and perforin. Further studies are required to unravel the role of latently infected CD8^+^CD4^- ^T cells in the pathogenesis of WD-MCF and particularly in the acute outcome of this disease.

## Competing interests

The authors declare that they have no competing interests.

## Authors' contributions

BD conceived and performed the experiments and wrote the manuscript; AV participated in the design of the study and coordination and helped wrote the manuscript. All authors read and approved the final manuscript.
